# Exploring the difference in xerogels and organogels through *in situ* observation

**DOI:** 10.1098/rsos.170492

**Published:** 2018-01-31

**Authors:** Binglian Bai, Zhiming Li, Haitao Wang, Min Li, Yukihiro Ozaki, Jue Wei

**Affiliations:** 1 Key Laboratory for Automobile Materials (JLU), Ministry of Education, Jilin University, Changchun, People's Republic of China; 2 College of Physics, Jilin University, Changchun, People's Republic of China; 3 Department of Chemistry, School of Science and Technology, Kwansei Gakuin University, 2-1 Gakuen, Sanda 669-1337, Japan

**Keywords:** *In situ* observation, organogels, xerogels, lytropic liquid crystals

## Abstract

Solvent–gelator interactions play a key role in mediating organogel formation and ultimately determine the physico-chemical properties of the organogels and xerogels. The ethanol organogels of 1,4-bis[(3,4,5-trihexyloxy phenyl)hydrazide]phenylene (TC6) were investigated *in situ* by FT-IR, Raman and fluorescence spectra, and XRD, and it was confirmed that the intermolecular interaction and aggregation structure of TC6 ethanol organogels were quite different from those of xerogels. Simultaneously, unprecedented phase transition from organogel to suspension upon heating was observed in ethanol organogel, and the suspension phase exhibited lytropic liquid crystalline behaviour with a rectangular columnar structure. This study may open the possibility to design new gelators with a new dimension of versatility.

## 1. Background

Low-molecular-weight organogels (LMOGs) are composed of three-dimensional networks due to the self-assembly of organogelators through non-covalent interactions (such as hydrogen bonding, π–π stacking and van der Waals interaction) and a large amount of solvents therein [1,2]. Organogels are of particular interest because of their unique attributes of non-polymeric nature and sensitive response to external stimulation, such as temperature, light and metal ions [3–9]. Thereby, organogels are considered as ‘smart’ materials and have found diverse applications [10–12]. It is essentially important to have a clear understanding of their aggregation structure for novel molecular design of organogelators and their application. However, the packing arrangements of gelators in organogels are unknown in most cases, although different methods, such as SEM, XRD and atomic force microscopy (AFM), have been used for characterization of organogels. One of the empirical approaches towards the solution of this problem was to assume that the molecular packing in the organogel state may be reflected by the xerogel properties. Thus, xerogels were usually investigated in order to infer the structure of organogels.

Solvent played an important role in the formation of an organogel and the determination of their physico-chemical properties. It was demonstrated that morphologies of gels could be modified by solvent conditions (polarity, concentration and pH condition) for the same gelator or a slightly modified structure of the same type of gelator for the same solvent [13–15]. It was reported that solvent might be involved in the formation of organogels [16–20], for example, solvent–gelator interaction was confirmed in sugar-based organogels by field-cycling NMR relaxometry [17]. Whitten and co-workers observed the sol-to-gel conversion of a cholesterol derivative, and their AFM results showed that solvent molecules are permeated inside the bundles, either within the elemental fibres or in ‘channels’ between them [18]. Sakurai and co-workers reported that the alcohol solvents with a longer alkyl chain are involved inside the gel fibre because the longer alkyl alcohols were more favourable to interact with the dodecyl chains of the gelator [19]. Furthermore, they reported that the *p*-xylene solvent molecules are incorporated into the gel fibre of methyl 4,6-*O*-benzylidene- α-d-mannopyranoside which was stabilized by the π–π interaction between the aromatic moiety and the *p*-xylene solvent [20]. However, until now, whether the structure of organogel is the same as that of xerogel in the case of organic solvent participating in the formation of organogel still has not been paid much attention.

Many LMOGs have been reported to be polymorphic, i.e. more than one molecular packing arrangement can be identified when forming fibrillar networks in gels from different solvents [21,22]. For example, Weiss and co-workers have reported that the CCl_4_ gel phases of a series of low-molecular mass organogelators (HSN-*n*) exhibited gel-to-gel phase transitions, which were considered to be due to the changes in the molecular packing of the HSN-*n* within the fibres [23].

**Scheme 1.**
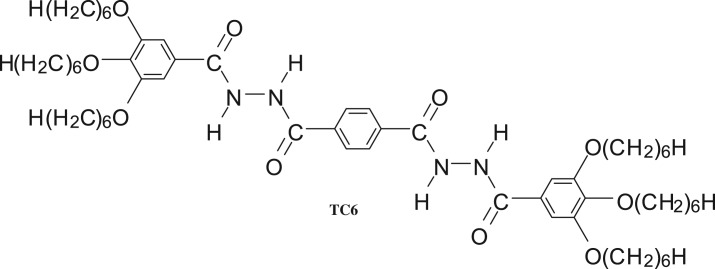
Chemical structure of compound TC6.

In this paper, the organogel properties of 1,4-bis[(3,4,5-trihexyloxy phenyl)hydrazide]phenylene (TC6, Scheme 1) in ethanol (EtOH) was fully investigated *in situ* by FT-IR, Raman and fluorescence spectroscopy, and XRD. The results indicated that the intermolecular interaction and aggregation structure of TC6 EtOH organogels were quite different from those of xerogels. Simultaneously, a new organogel-to-suspension phase transition was observed in ethanol organogel, and the results indicated that the suspension exhibited lytropic liquid crystalline behaviour with a rectangular columnar structure.

## 2. Material and methods

### 2.1. Materials

The compound TC6 was synthesized in our laboratory, and its chemical structure was confirmed by FT-IR, ^1^H NMR spectroscopy and elemental analysis. The synthesis details were reported elsewhere [24].

### 2.2. Characterization

The xerogels were obtained by freezing at −50°C and pumping the organogel of TC6 in 1,2-dichloroethane (DCE) and EtOH for 12 h, except that the xerogels used to obtain the time-dependent IR spectra were dried at room temperature. FT-IR spectra were recorded at a spectral resolution of 4 cm^−1^ with a Thermo Scientific Nicolet 6700 spectrometer. The Raman spectra of TC6 were measured at room temperature at a spectral resolution of 4 cm^−1^ using a Raman microscope (HR800, Horiba), with the excitation wavelength of 514.5 nm. The laser power at the sample point was 100 µW. The photoluminescence (PL) was measured on a PerkinElmer LS 55 spectrometer. XRD was carried out with a Rigaku RINT2100 X-ray diffractometer. The thermal properties of the compounds were investigated by differential scanning calorimetry (DSC) with a PerkinElmer Pyris DSC instrument. The rate of heating was 10°C min^−1^. Optical textures were observed by polarizing optical microscopy (POM) using a Leica DMLP microscope equipped with a Leitz 350 heating stage. ^1^H NMR spectra were recorded with a Bruker Avance 500 MHz spectrometer, using chloroform-*d* as a solvent and tetramethylsilane as an internal standard (*d* = 0.00). During the DSC, FT-IR, temperature-dependent FT-IR, fluorescence spectra and XRD measurements, the concentration of TC6 organogels in EtOH was 1.42%, and in DCE was 0.84%.

## 3. Results and discussion

### 3.1. Intermolecular interaction

**Figure 1.**
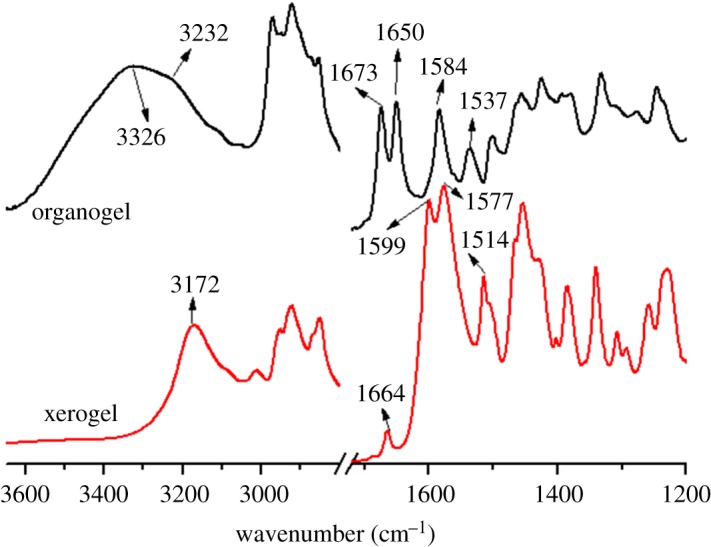
FT-IR spectra of TC6 xerogel and organogel from EtOH.

In our previous work, we reported the self-assembly behaviour of TC6, and its stable organogelation in many solvents, such as DCE and EtOH [24]. To study the intermolecular interactions in EtOH organogel and xerogel, FT-IR spectra of TC6 organogel and xerogel in EtOH were measured, and the results are shown in figure 1. (Hereafter, mainly the spectral regions of 3600–3000 and 1700–1500 cm^−1^ are discussed, because the spectra in these regions may provide the information about intermolecular interactions.) The FT-IR spectra of TC6 EtOH xerogel are similar to that in the crystalline phase [25]. Bands at 3172 and 3012 cm^−1^ are assigned to the NH stretching mode of the hydrazide group and the CH stretching mode of the aromatic ring, respectively [25]. Bands at 2954 and 2870 cm^−1^ arise from the asymmetric (*ν*_αs_) and symmetric (*ν*_s_) stretching vibrations of the CH_3_ groups, whereas the bands at 2922 and 2852 cm^−1^ arise from the asymmetric (*ν*_αs_) and symmetric (*ν*_s_) stretching vibrations of the CH_2_ groups. A weak band at 1664 cm^−1^ and a strong one at 1577 cm^−1^ may be attributed to amide I (the band at 1577 cm^−1^ may superimpose certain bands due to *ν*(C═C) [25]). IR bands at 1599 and 1514 cm^−1^ may be assigned to the *ν*(C═C) of phenyl ring and amide II, respectively [25]. The observed *ν*(N─H) (3172 cm^−1^) and amide I (1664 and 1577 cm^−1^) clearly indicate that in the EtOH xerogel, almost all the ─NH groups in TC6 are associated with the ─C═O groups via ─NH⋯O═C─H-bonds [26]. It can be supported by the fact that the *ν*(N─H) and *ν*(C═O) bands become weaker and shift towards higher wavenumbers upon heating, which is typical for H-bonded ─NHs and ─C═Os [25]. However, in the EtOH organogel, a strong band at 3326 cm^−1^ may be attributed to the bonded *ν*(OH) [27]. The *ν*(N─H), amide I shifts to higher wavenumbers obviously, appearing at 3232 cm^−1^ (*ν*(N─H)), 1673 and 1650 cm^−1^ (amide I), respectively, and the amide II shifts from 1514 to 1537 cm^−1^. It can be seen that the intermolecular hydrogen bonding of the organogel in EtOH is much weaker than that of the xerogel. Simultaneously, a band due to *ν*(C═C) of phenyl ring at 1599 cm^−1^ in the xerogel shifts to 1584 cm^−1^ in the organogel, indicating the π–π interaction of the organogel in EtOH is also much weaker than that of the xerogel [25]. Based on the above results, we may be able to conclude that EtOH molecules interact with TC6 through intermolecular hydrogen bondings, which weaken the intermolecular interaction among TC6.

**Figure 2.**
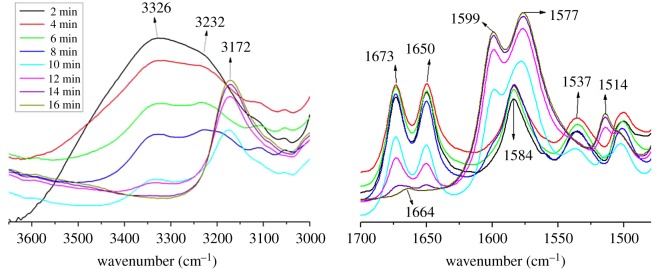
Time-dependent FT-IR spectra of TC6 organogel in EtOH at room temperature.

Time-dependent FT-IR spectra of EtOH organogel at room temperature were measured to investigate the spectral changes from organogel to xerogel (figure 2). With the ethanol volatilization, the band at 3326 cm^−1^ gradually becomes weak and finally disappears, and no spectral shift was observed in this process. Therefore, it is very likely that the band at 3326 cm^−1^ is attributed to the bonded ─OH stretching vibrations of EtOH molecules [27]. The bands due to *ν*(N─H), amide I, *ν*(C═C) and amide II all exhibit notable shifts, i.e. from 3232 cm^−1^ (*ν*(N─H)), 1673 and 1650 cm^−1^ (amide I), 1584 cm^−1^ (*ν*(C═C)) and 1537 cm^−1^ (amide II) in the organogel to 3172 cm^−1^ (*ν*(N─H)), 1664 and 1577 cm^−1^ (amide I), 1599 cm^−1^ (*ν*(C═C)) and 1514 cm^−1^ (amide II) in the xerogel, respectively, indicating that the intermolecular hydrogen bonding and π–π interaction remarkably increase from organogel to xerogel.

**Figure 3.**
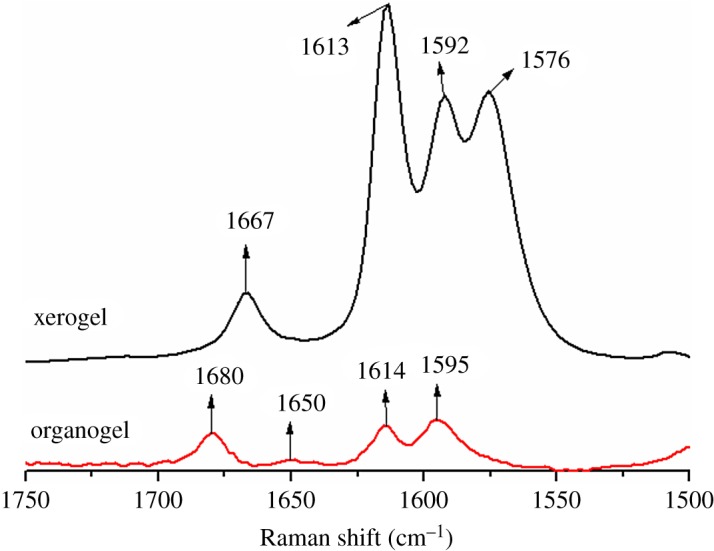
Raman spectra in the region of 1750–1500 cm^−1^ of TC6 xerogel and organogel from EtOH.

Raman spectra in the region of 1750–1500 cm^−1^ of TC6 organogel and xerogel in EtOH are shown in figure 3. Characteristic bands of amide I shift from 1667 and 1576 cm^−1^ in EtOH xerogel [25] to 1680 and 1650 cm^−1^ in EtOH organogel, indicating that the intermolecular hydrogen bonding is weaker in EtOH organogel than the corresponding xerogel.

**Figure 4.**
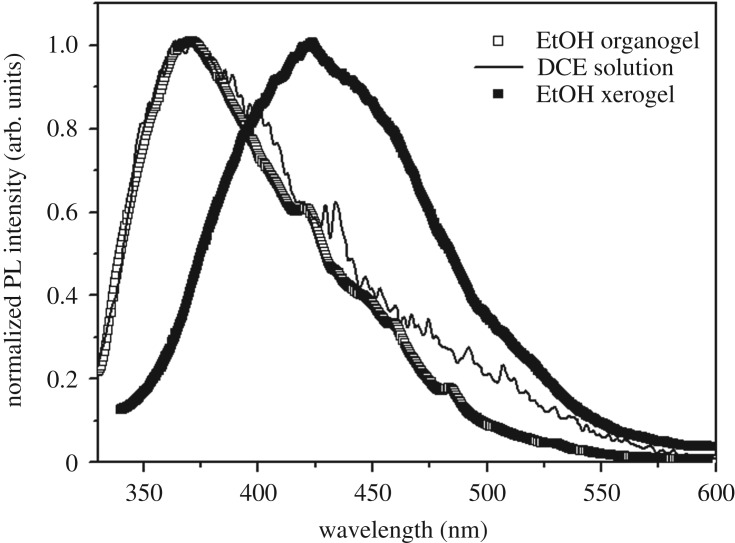
The normalized fluorescence emission of TC6 organogel and xerogel in EtOH and solution (7.2 × 10^−5^ mol l^−1^) in DCE.

As shown in figure 4, the maximum emission peak is located at 422 nm in the TC6 xerogel from EtOH, which is the same as that in DCE organogel and xerogel (electronic supplementary material, figure S5), whereas the EtOH organogel shows an emission peak at 368 nm, which is the same as that in the DCE solution (7.2 × 10^−5^ mol l^−1^) arising from TC6 monomer. It may be because the organogels self-assemble mainly through the intermolecular hydrogen bonding between EtOH molecules and TC6, and the intermolecular distances as well as the degree of molecular freedom of TC6 molecules in organogels are greater than those of xerogels, so the EtOH organogel exhibits monomeric emission characteristic. Whereas, the TC6 molecules can self-assemble through the intermolecular hydrogen bonds between ─C═O⋯HN─ in EtOH xerogel, the intermolecular distances decrease and the aggregation induced a more planar structure in the xerogels state, thus the maximum emission peak has a large red shift. The more planar structure in the xerogels state is evidenced by the absorption band of the xerogel with a large red shift and spectrum broadening relative to that in solution and organogel (electronic supplementary material, figure S8).

In addition, in order to study the effect of temperature on the intermolecular interaction, the temperature-dependent fluorescence and FT-IR spectra of TC6 organogels in EtOH were measured. The temperature-dependent fluorescence emission of TC6 organogel in EtOH shows that the intensity of the fluorescence of TC6 in the gel state is almost unchanged as the temperature increases. When the temperature reaches to approximately 65°C (the organogel dissociated), the intensity of the emission is obviously decreased and it is almost non-fluorescent (electronic supplementary material, figure S11). It is probable that the heating dissociates the intermolecular hydrogen bonding between TC6 and EtOH in organogel, which is the main driving force supporting gel formation, and the degree of molecular freedom and the molecular twist increase, which induces the emission quenching.

**Figure 5.**
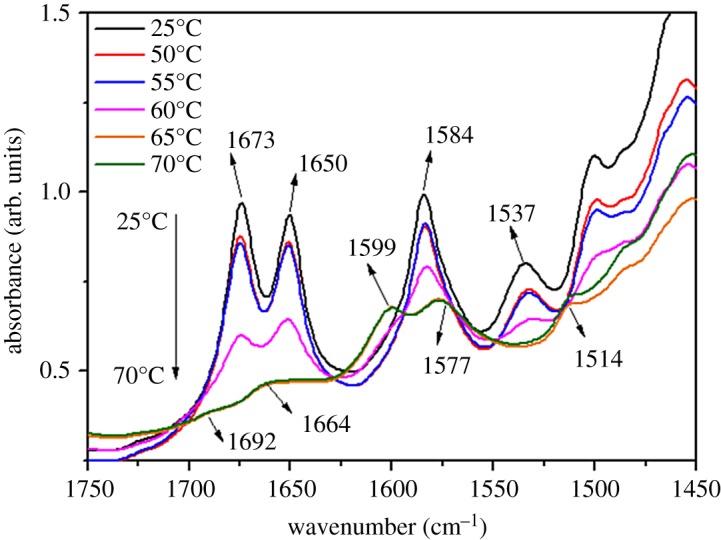
Temperature-dependent FT-IR spectra in the region of 1750–1450 cm^−1^ of TC6 organogels in EtOH.

Figure 5 displays temperature-dependent FT-IR spectra of TC6 organogels in EtOH. With increasing temperature, the characteristic absorption band of amide I, *ν*(C═C) of phenyl ring and amide II becomes weak and shifts from 1673 and 1650 cm^−1^ (amide I), 1584 cm^−1^ (*ν*(C═C)), 1537 cm^−1^ (amide II) in organogel to 1692, 1664 and 1577 cm^−1^ (amide I), 1599 cm^−1^ (*ν*(C═C)), 1514 cm^−1^ (amide II) at 65°C, suggesting the destruction of the intermolecular hydrogen bonding interaction between the solvent (EtOH)–gelator (TC6) interaction in organogel and the construction of gelator (TC6)–gelator (TC6) interaction with the increase of temperature to 65°C.

### 3.2. Aggregation structure

**Figure 6.**
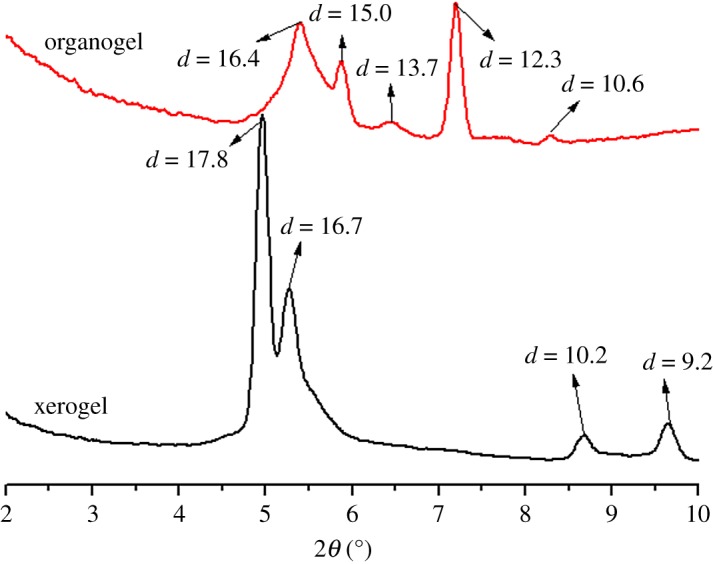
XRD profiles of the xerogels and organogels in EtOH.

To reveal the packing structures of organogels and xerogels in EtOH, XRDs were measured for the xerogels and organogels in EtOH (figure 6). The XRD pattern of the EtOH xerogel of TC6 consists of four peaks at 17.8 (200), 16.7 (110), 10.2 (310) and 9.2 (400) Å, corresponding to a rectangular columnar packing (*a* = 35.6 Å, *b* = 19.0 Å). By contrast, the XRD pattern of the EtOH organogel of TC6 yields five diffractions at 16.4, 15.0, 13.7, 12.3 and 10.6 Å, which is wholly different from that of its EtOH xerogel.

### 3.3. Lytropic liquid crystallinity in EtOH organogels

**Figure 7.**
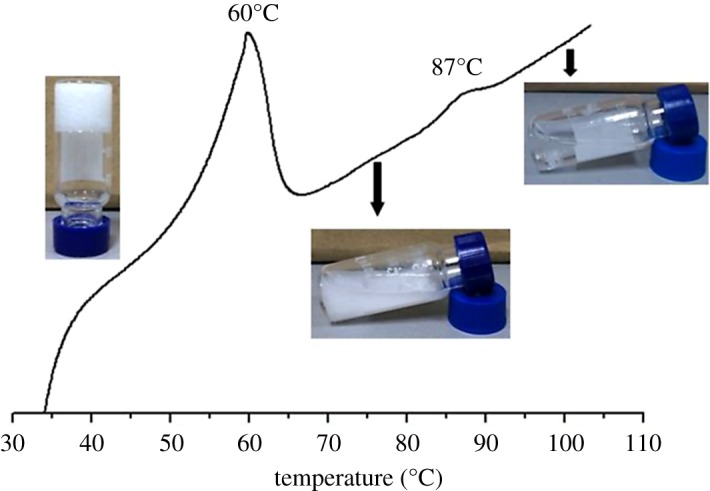
DSC curve of TC6 organogels in EtOH (1.42%) on the first heating run.

**Figure 8.**
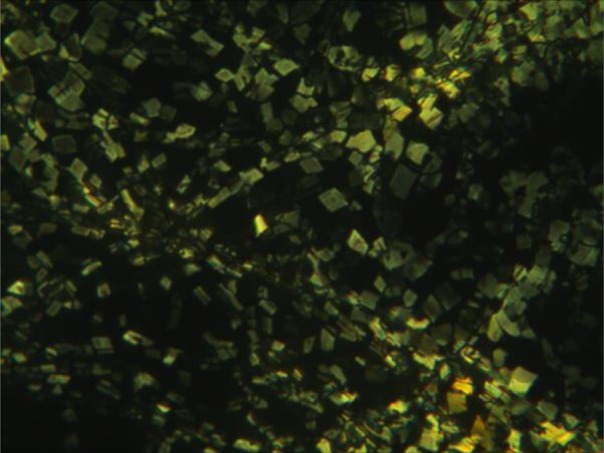
POM of TC6 EtOH organogels at 70°C (200×).

Interestingly, the TC6 organogel in EtOH exhibits two-step melting behaviour as shown in figure 7, i.e. a strong broad endothermic peak at approximately 60°C corresponds to organogel-suspension transition, correlating well with the melting temperatures (*T*_gel_) of the organogels as measured with the falling drop method, and the small peak at 87°C corresponds to suspension-sol transition. The suspension shows quadrilateral texture under POM (figure 8). The XRD pattern (electronic supplementary material, figure S15) of TC6 suspension in EtOH at 70°C consists of three peaks at 18.4 (200), 17.0 (110) and 7.6 (500) Å, corresponding to a rectangular columnar structure (*a* = 36.8 Å, *b* = 19.2 Å). It may be concluded that TC6 suspension (figure 7) in EtOH between 60°C and 87°C is lyotropic liquid crystalline state, which becomes isotropic solution at 87°C. The temperature-dependent FT-IR spectra of TC6 organogels in EtOH are shown in figure 5, the amide I appears at 1692 (free), 1664 (weak bonding) and 1577 (strong bonding) cm^−1^ at 65°C, indicating that the majority of the ─C═O are associated with the ─NH groups via N─H⋯O═C hydrogen bonding and others are free in lyotropic liquid crystalline state.

On the basis of the results described above, a possible representation of the structural changes of TC6 gel in EtOH can be proposed. In EtOH organogel, the ─OH groups of EtOH molecules could probably form intermolecular hydrogen bonding with its ─OH group, ─NH and ─C═O of TC6. Considering that the EtOH molecules are quite small and easy to insert among gelators, and then weaken the intermolecular hydrogen bondings between the ─C═O and ─NH group of TC6, the elemental fibrils of organogels, namely one-dimensional supramolecular chain, can self-assemble mainly through the intermolecular hydrogen bonding between EtOH molecules and TC6, as well as very weak intermolecular hydrogen bondings between the ─C═O and ─NH group. Thus, the intermolecular distance between TC6s increases and the EtOH organogel exhibits monomeric characteristic of its PL spectra. In its suspension, the intermolecular hydrogen bonding between TC6 and EtOH is destroyed, and the fibres as well as their three-dimensional networks of the organogel are dissociated, the TC6 molecules rearrange through the intermolecular hydrogen bondings between the ─C═O and ─NH group to give a rectangular columnar mesophase.

In the EtOH xerogel with no EtOH at all, TC6 molecules self-assemble through the intermolecular hydrogen bonds between ─C═O⋯HN─ to form one-dimensional supramolecular chain and further to form fibrous bundles showing a rectangular columnar structure.

## 4. Conclusion

The organogel behaviour of TC6 in EtOH was fully investigated *in situ* by FT-IR, Raman, and fluorescence spectra, XRD and DSC. The results of FT-IR, Raman and fluorescence spectra showed that the elemental fibrils of organogels self-assemble mainly through the intermolecular hydrogen bonding between EtOH molecules and TC6 and very weak intermolecular hydrogen bondings between the ─C═O and ─NH groups, whereas the elemental fibrils of xerogels self-assemble mainly through the intermolecular hydrogen bonding between the ─C═O and ─NH group. The XRD results suggested that the aggregation structure of organogels are completely different from those of the xerogels, which indicated that the solvent can adjust the interaction of molecular aggregates, and then regulated the aggregated structure. Simultaneously, the unprecedented lytropic liquid crystal with a rectangular columnar structure was observed after the organogel collapsed. The intermolecular hydrogen bondings between the ─C═O and ─NH groups were the major driving forces for the formation of the mesophase.

This study confirmed that the role of the solvent was very important in organogel formation and the properties of xerogel properties sometimes cannot reflect those of organogel. And it also identified the heretofore lytropic liquid crystals in EtOH organogels and the observation of the lytropic liquid crystals in organogel opened the possibility to design new gelators with a new dimension of versatility.

## Supplementary Material

Supporting information is located at step 6–file unload

## Data accessibility

Data used in this paper are available in the electronic supplementary material.
