# Supplementary material for: Exploring the difference in xerogels and organogels through in situ observation
Source: R Soc Open Sci. 2018 Jan 31;5(1):170492. doi: 10.1098/rsos.170492 (PMC5792869; doi:10.1098/rsos.170492)
Supplement: Supporting information is located at step 6–file unload [file rsos170492supp1.doc]

**Supporting information**

Mainly including (FT-IR, Raman, PL spectra, DSC and XRD of TC6 from DCE, ^1^H NMR, Absorption, PL spectra and DSC in EtOH)

**Exploring the difference in xerogels and organogels through in-situ observation**

Binglian Bai^a b^, Zhiming Li^b^, Haitao Wang^a^, Min Li*^a^, Yukihiro Ozaki^c^, Jue Wei*^b^

*^a^ Key Laboratory for Automobile Materials (JLU), Ministry of Education, Jilin University, Changchun, PR China,* [*minli@jlu.edu.cn*](mailto:minli@jlu.edu.cn)*; ^b^ College of Physics, Jilin University, Changchun, PR China,* [*weijue@jlu.edu.cn*](mailto:weijue@jlu.edu.cn)*; ^c^ Department of Chemistry, School of Science and Technology, Kwansei Gakuin University, 2-1 Gakuen, Sanda 669-1337, Japan,* [*ozaki@kwansei.ac.ip*](mailto:ozaki@kwansei.ac.ip)*.*


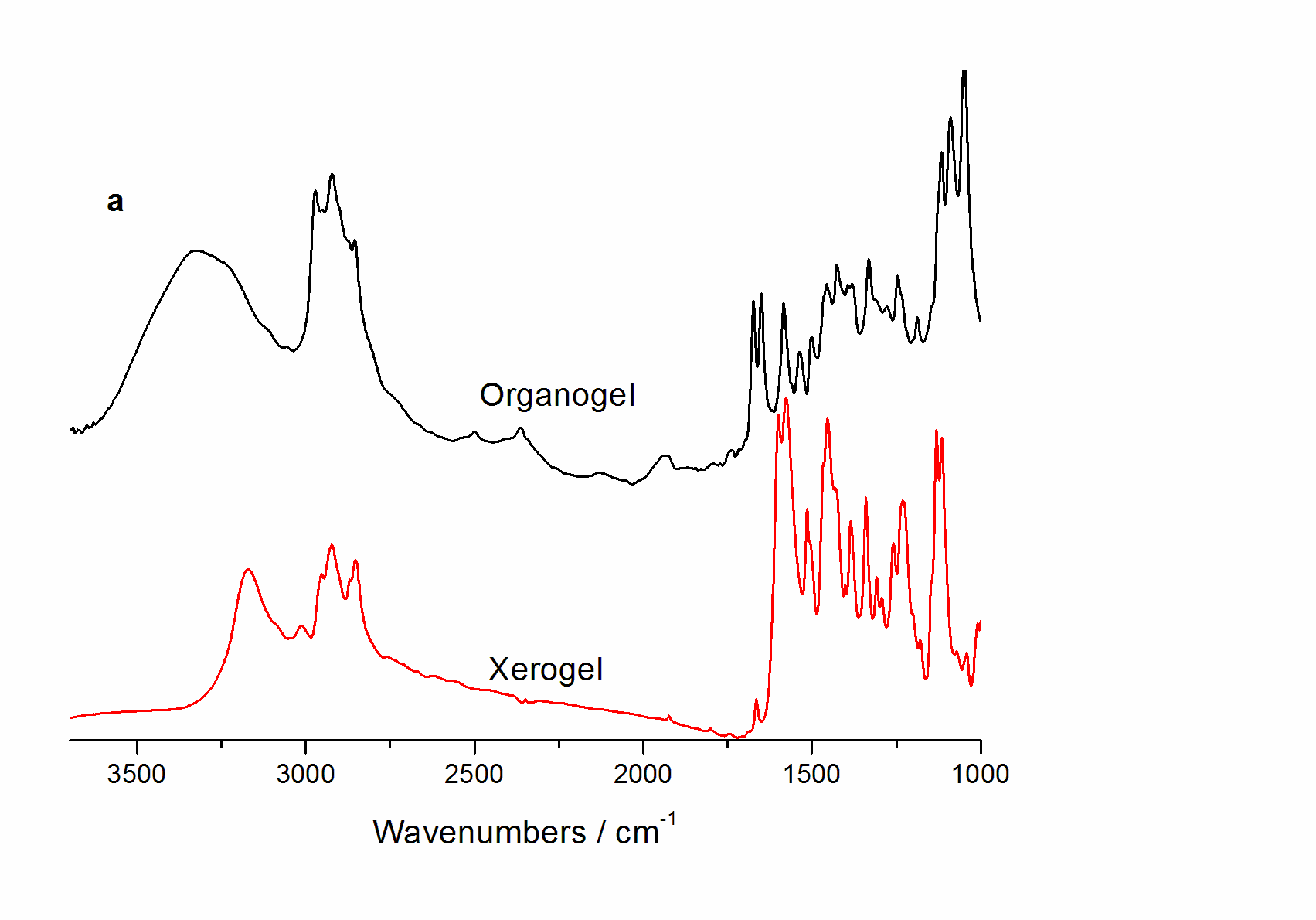


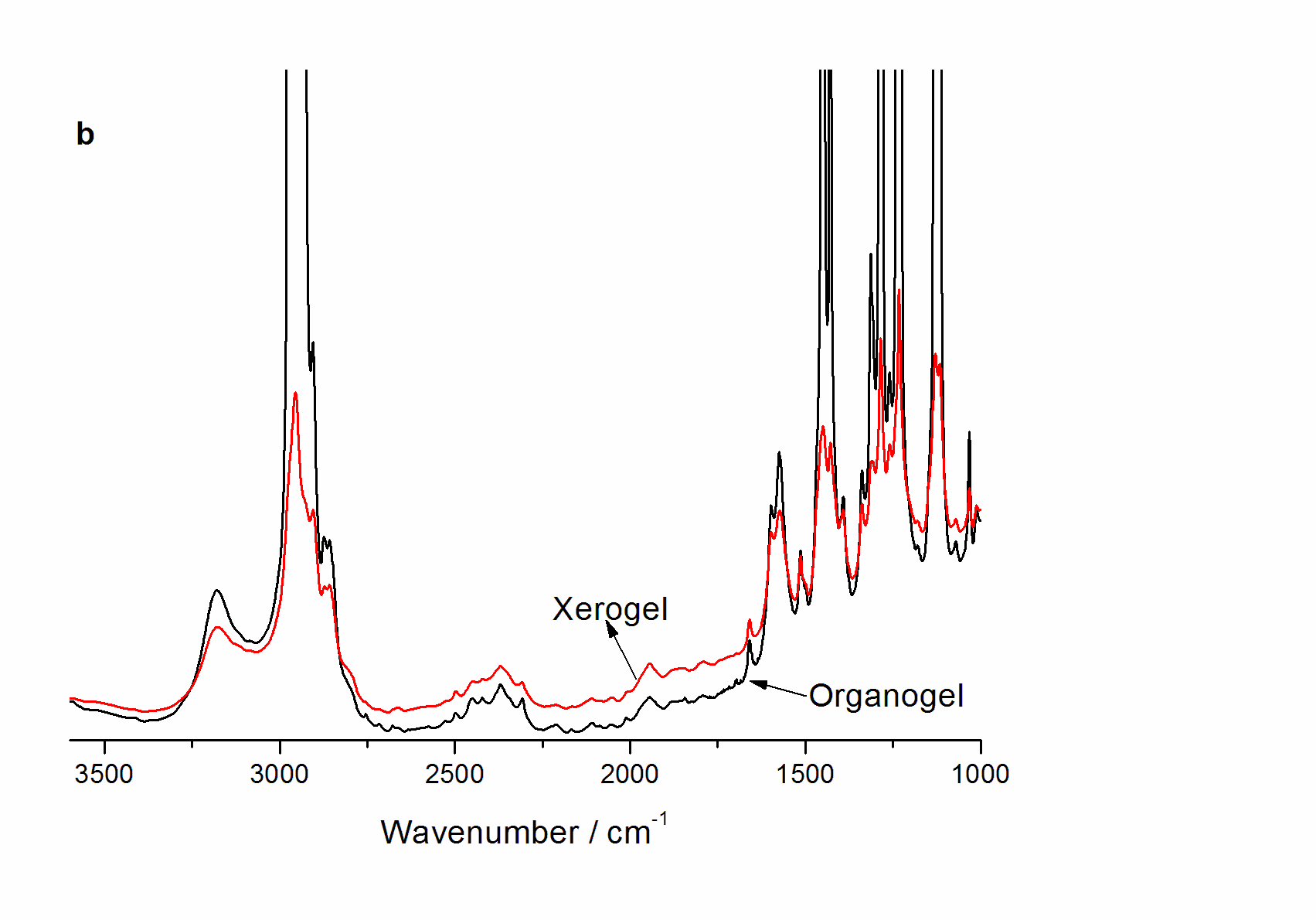


Figure S1 FT-IR spectra of TC6 xerogel and organogel from (a) EtOH and (b) DCE (in the region 3500-1000 cm^-1^).


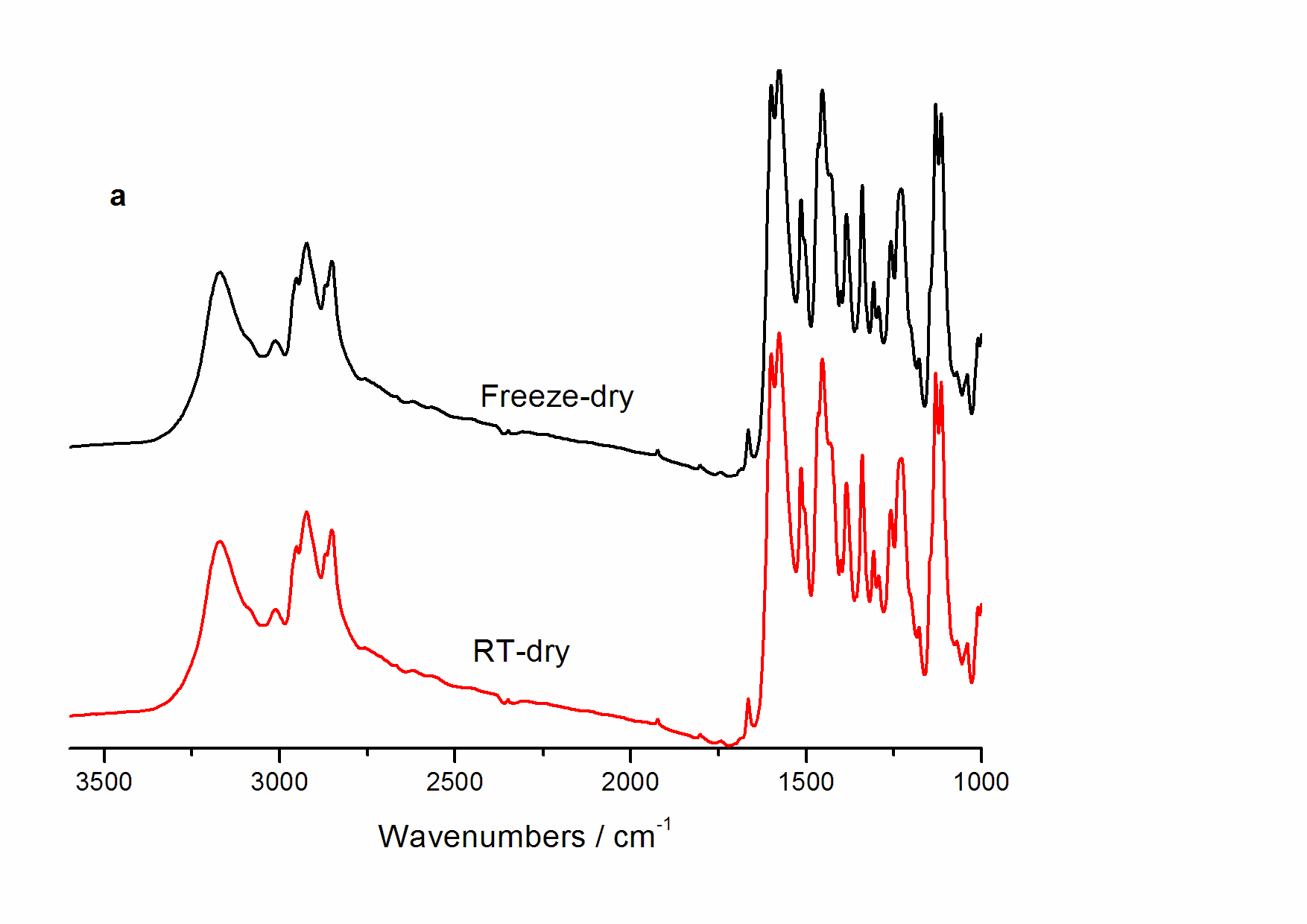


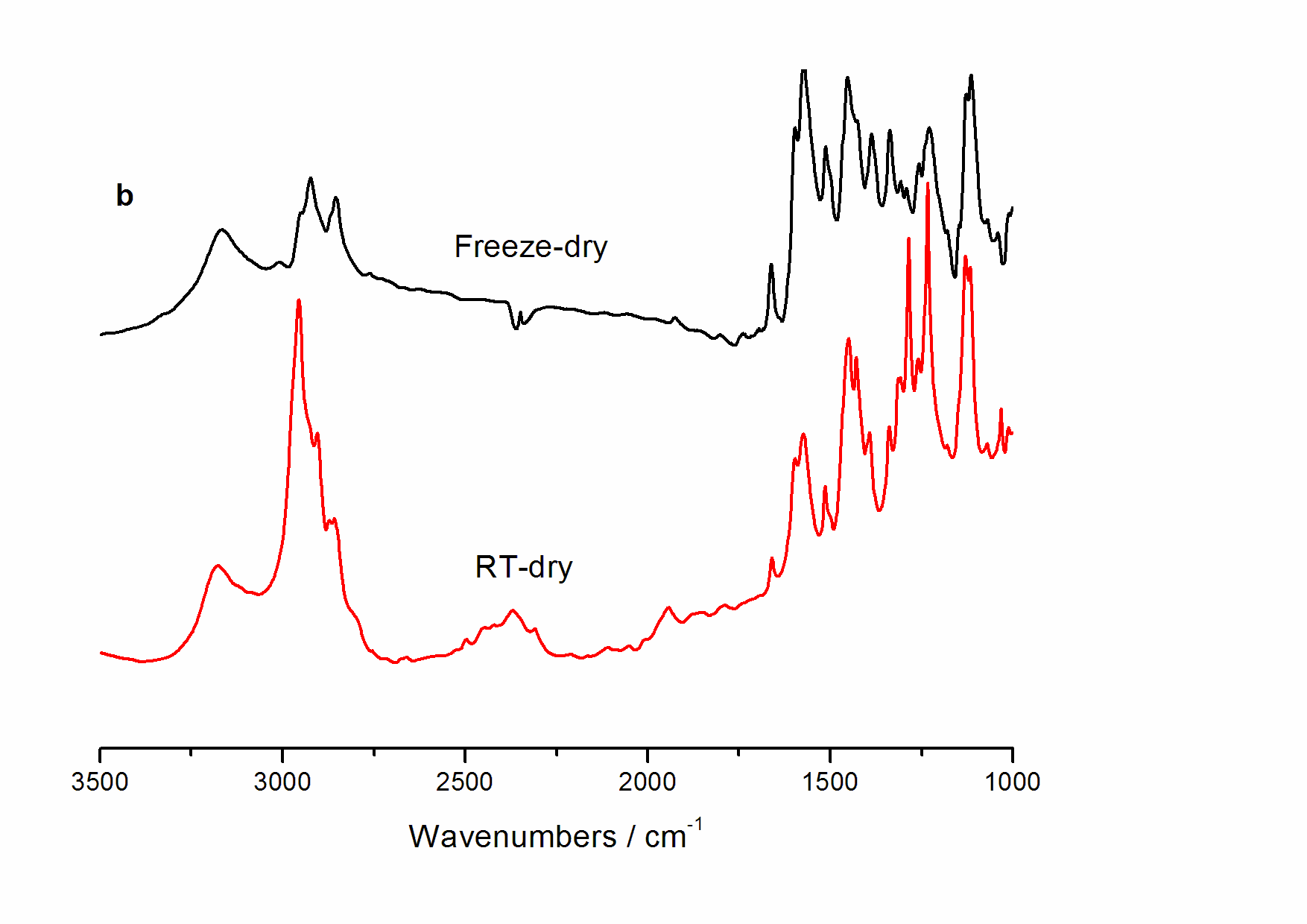


Figure S2 FT-IR spectra of TC6 xerogel (freeze-dry—the xerogel was dry at -50℃, and RT-dry—the xerogel was dry at room temperature) from (a) EtOH and (b) DCE.


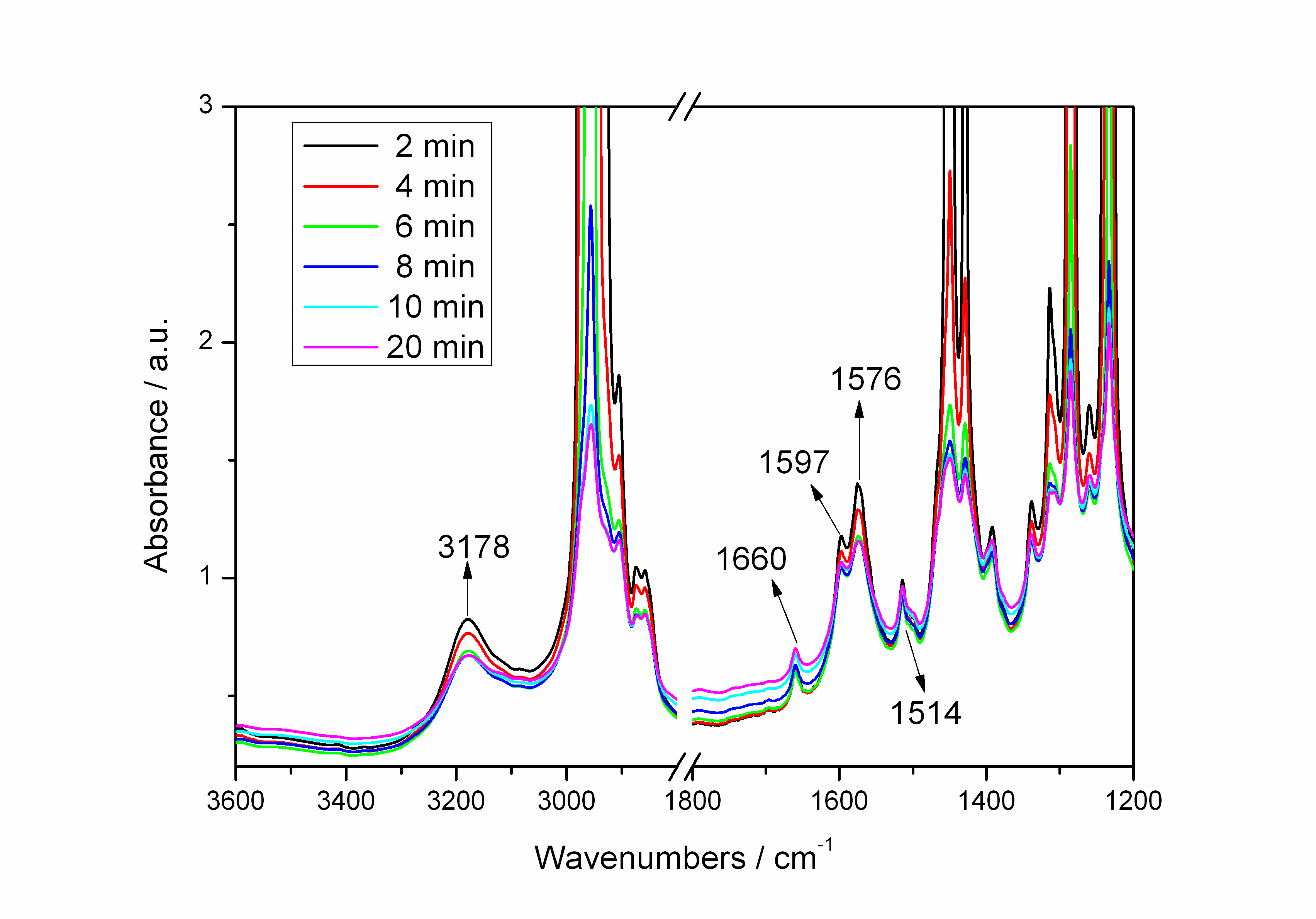


Figure S3 Time-dependent IR spectra of TC6 organogel from DCE.


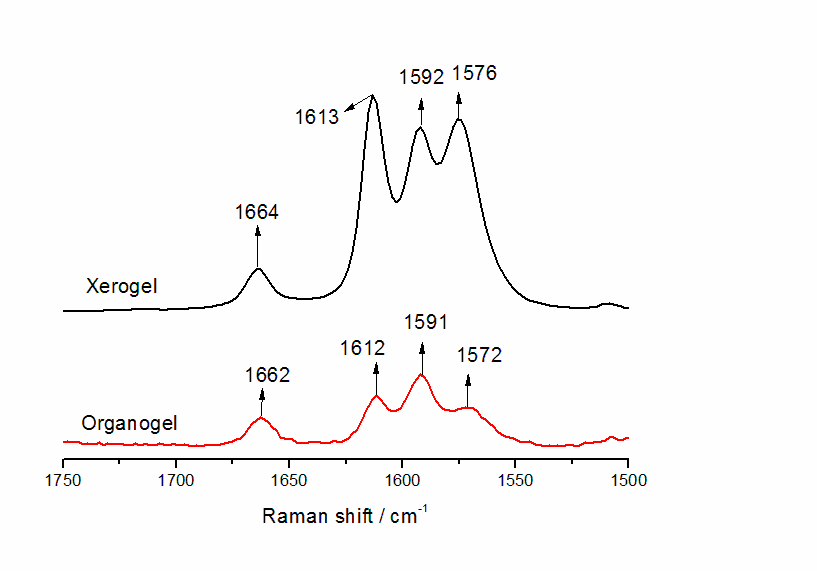


Figure S4　Partial Raman spectra of TC6 xerogel and organogel from DCE.


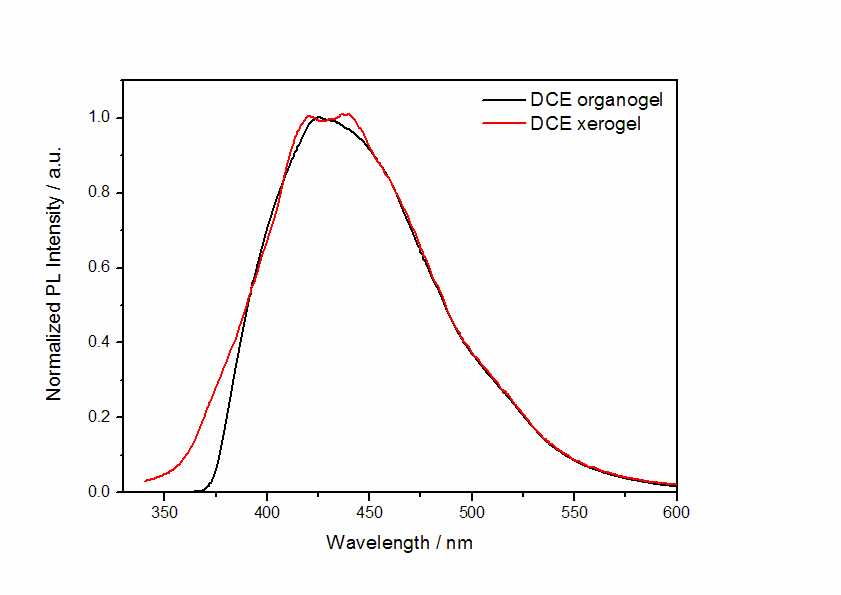


Figure S5 The normalized fluorescence emission of TC6 organogel and xerogel in DCE.


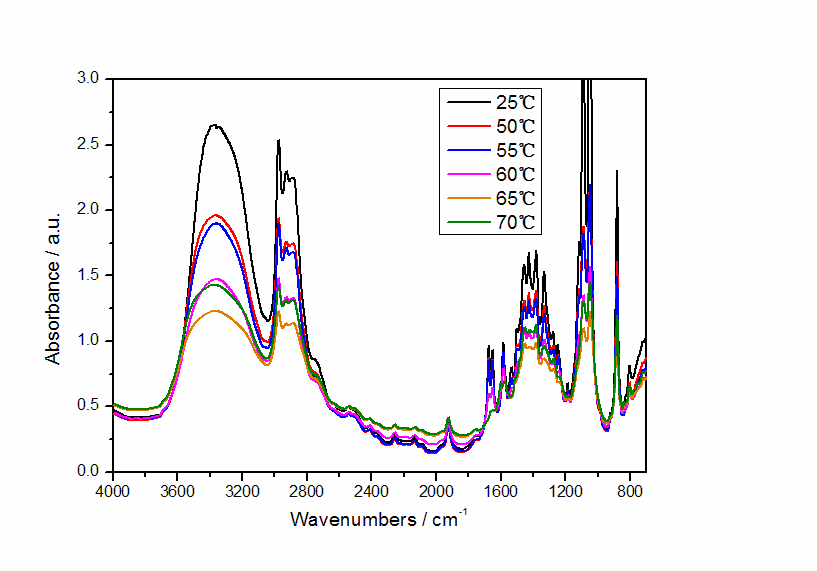


Figure S6 The temperature-dependent FT-IR spectra of TC6 organogels in EtOH (the enlarged spectra in the region of 1750-1450 cm^-1^ was shown in Figure 5).


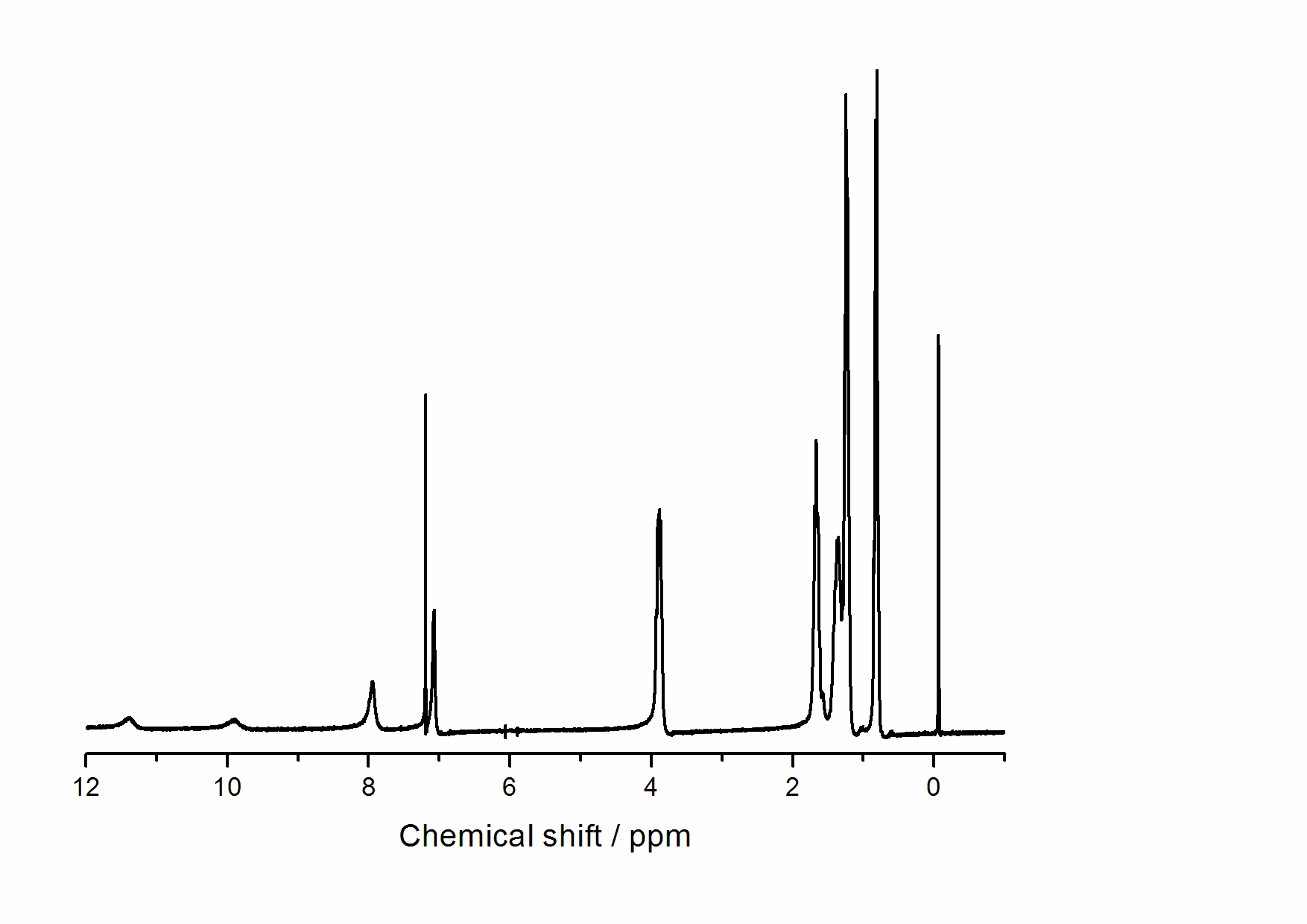


Figure S7 ^1^H NMR spectrum of **TC6** xerogel from EtOH in CDCl_3_ (No EtOH molecules was observed in xerogel).


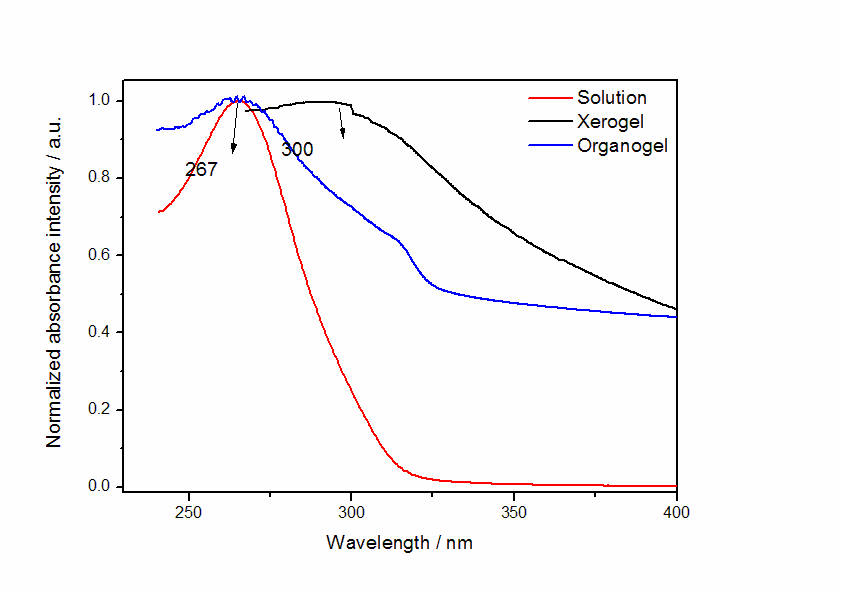


Fig. S8 Absorption spectra of TC6 organogel (0.37 wt%), xerogel and solution (1×10^-7^ mol/L) in EtOH.


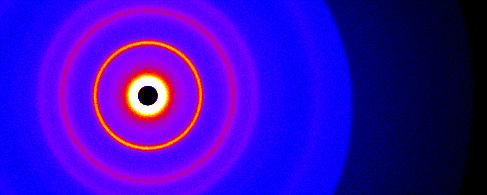

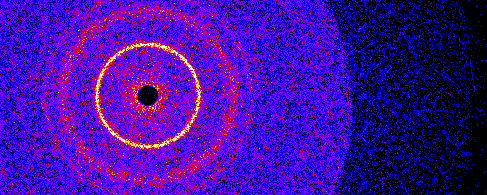


a b


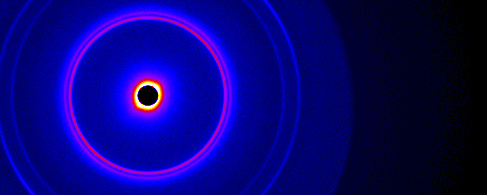

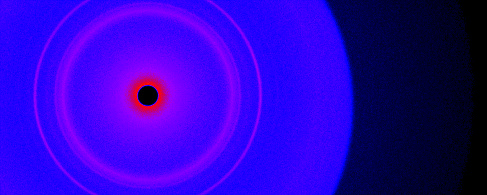


c d

Figure S9 The SAXRD patterns of the (a) xerogels and (b)organogels in the DCE, and (c) xerogels and (d)organogels in the EtOH.


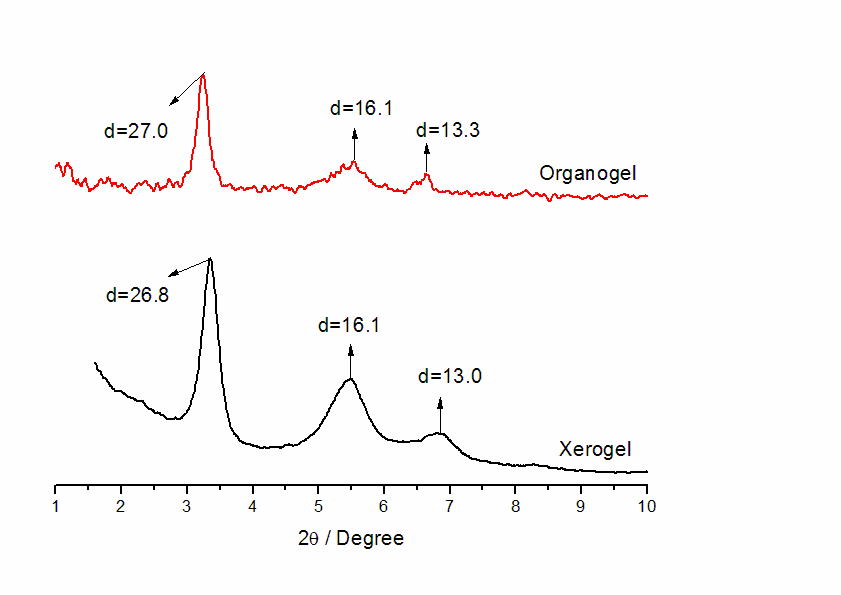


Figure **S10** The XRD profiles of the xerogels and organogels in the DCE.


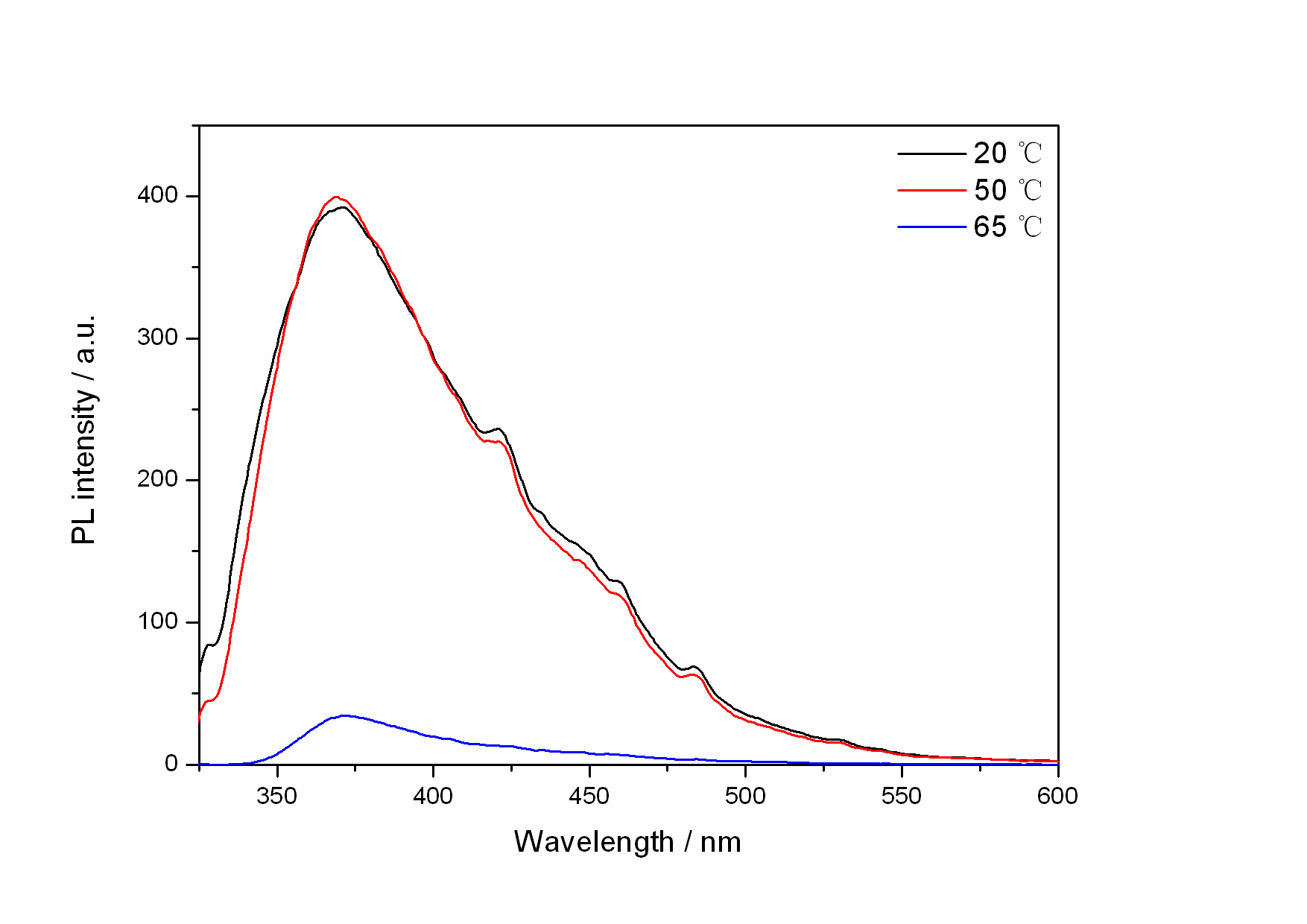


Fig. S11 Temperature-dependence of the fluorescence emission of TC6 organogel in EtOH.


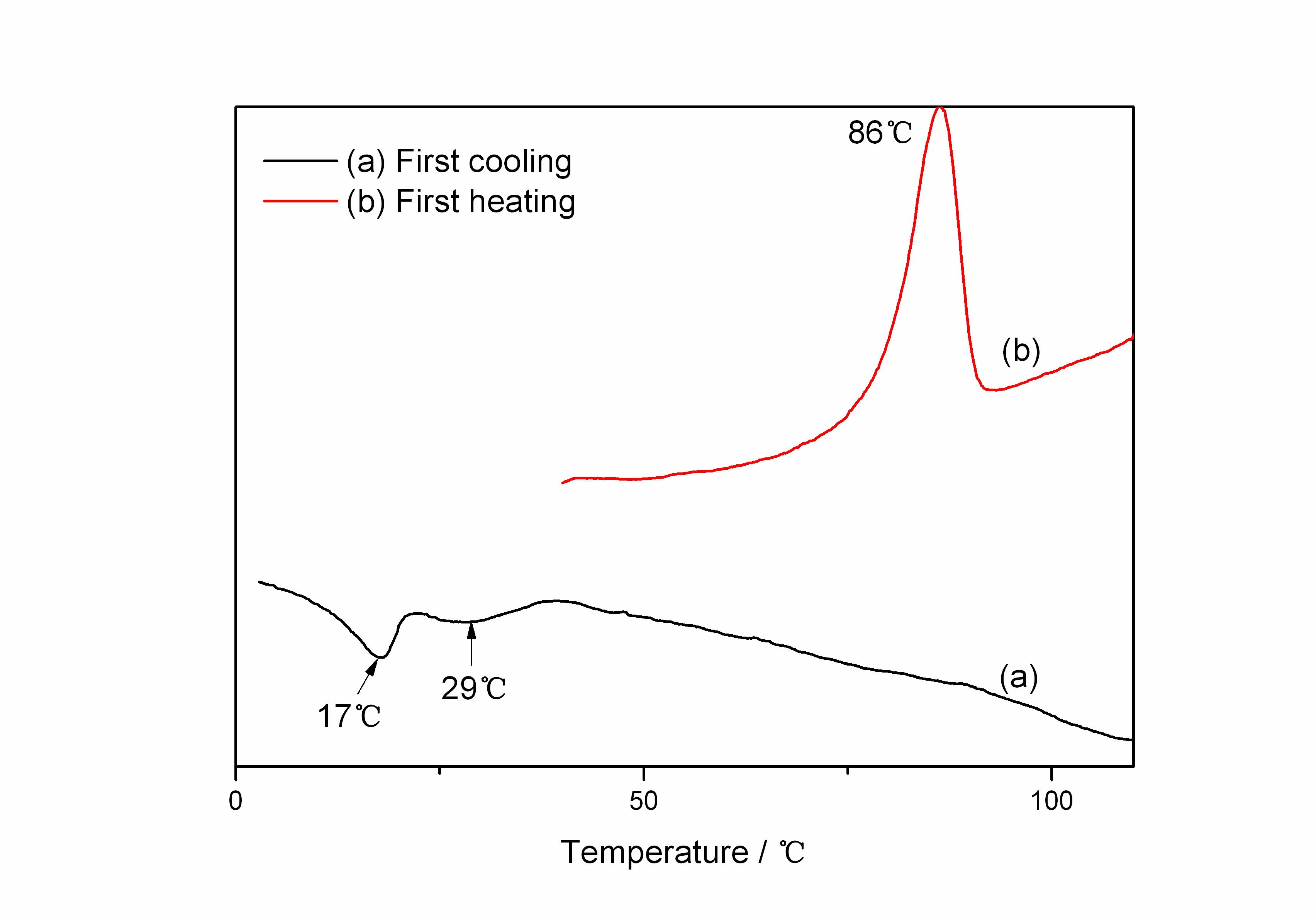


Fig. S12 DSC curve of TC6 organogels in DCE (0.84%) on the first heating and cooling run.


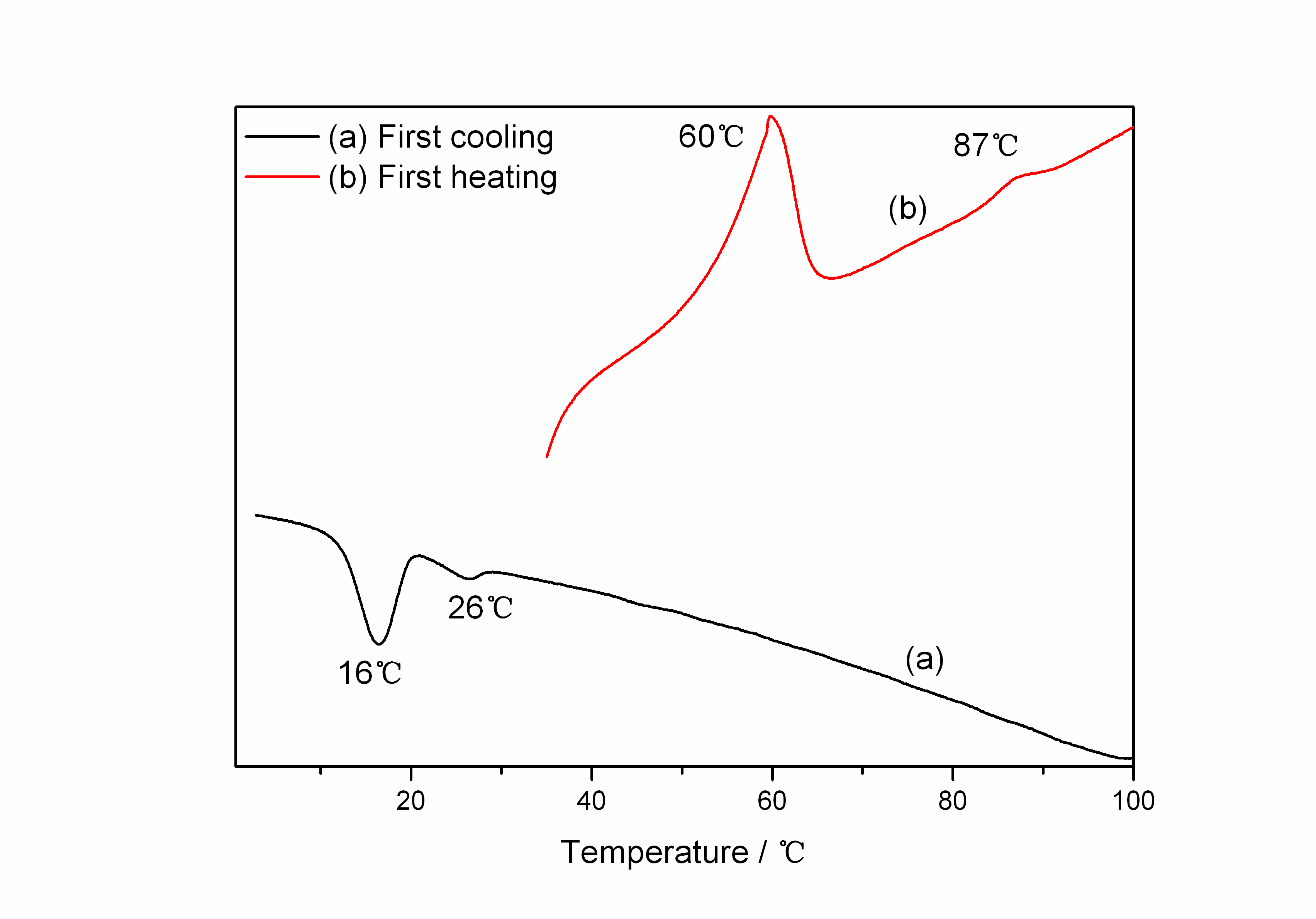


Fig. S13 DSC curve of TC6 organogels in EtOH (1.42%) on the first heating and cooling run.


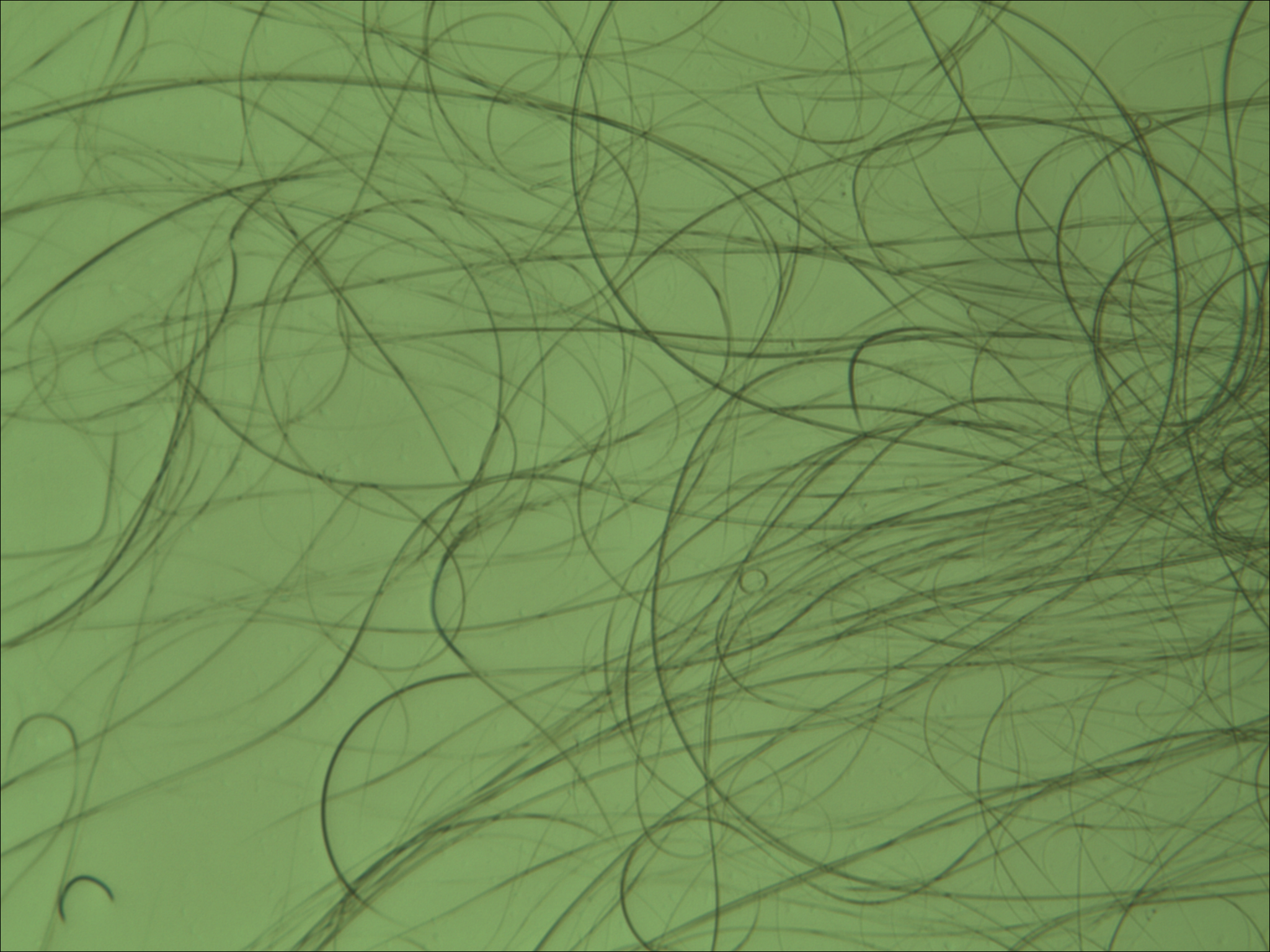

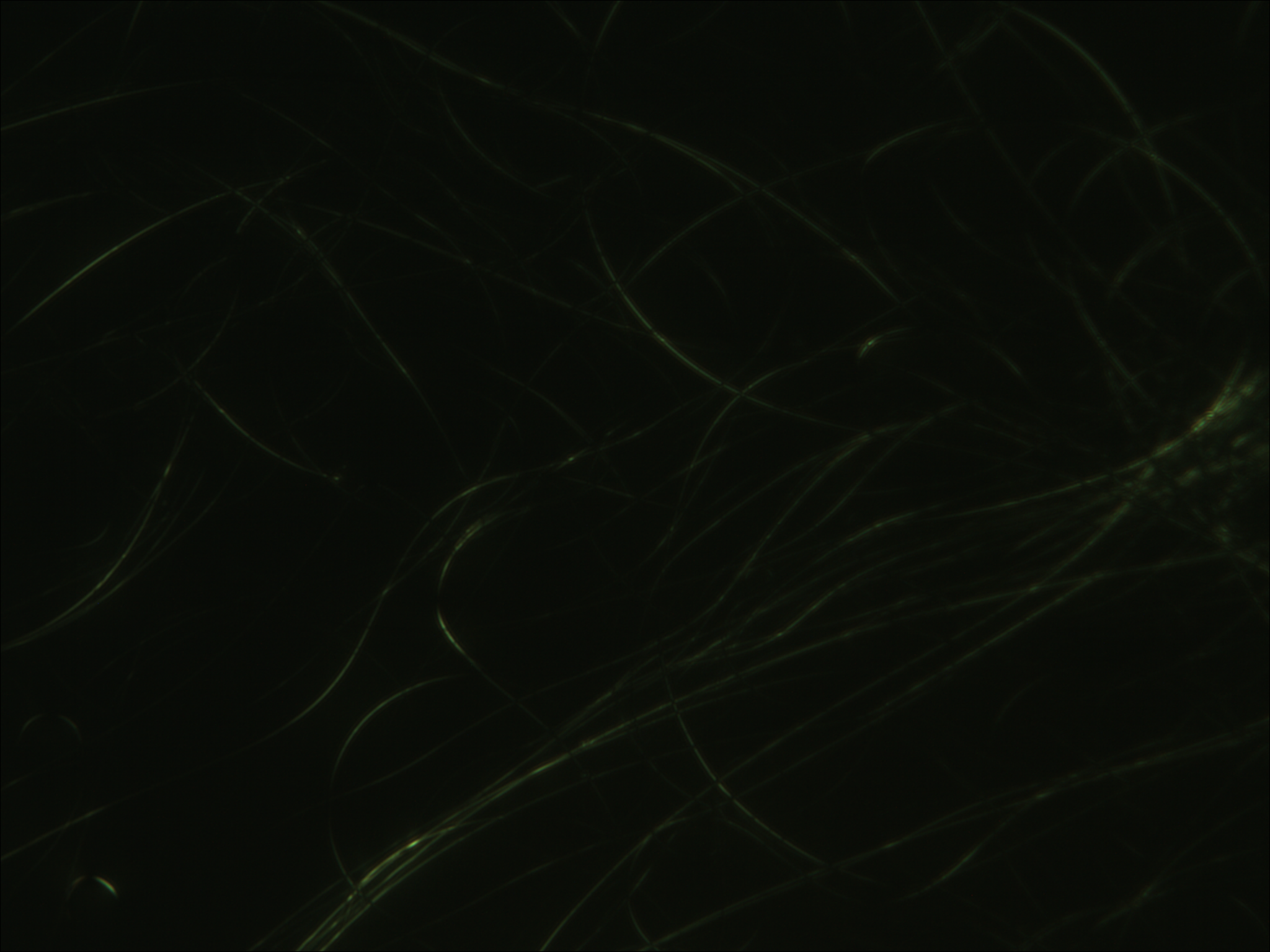


Figure S14 Optical microphotographs and POM of TC6 EtOH organogels (200 ×).


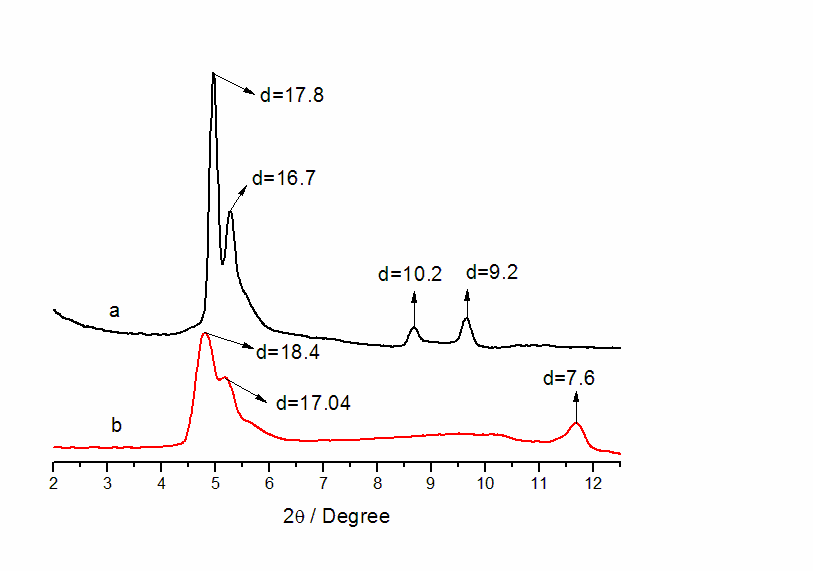


Fig. S15 The XRD profiles of the (a) xerogels and (b) organogels after heated to 70 ℃ in the EtOH.
